# 4234 Association of age at menopause with incident heart failure in the Southern Community Cohort Study

**DOI:** 10.1017/cts.2020.109

**Published:** 2020-07-29

**Authors:** Mindy Pike, Elvis A. Akwo, Cassianne Robinson-Cohen, Melissa Wellons, William Blot, T. Alp Ikizler, Thomas J. Wang, Deepak K. Gupta, Loren Lipworth

**Affiliations:** 1 Vanderbilt University Medical Center

## Abstract

OBJECTIVES/GOALS: Early age at menopause has been linked to increased risk of cardiovascular disease; however, there is limited evidence for a relationship between early menopause and heart failure (HF). We examined whether early menopause is associated with incident HF among women in the southeastern United States. METHODS/STUDY POPULATION: The Southern Community Cohort Study enrolled ~86,000 low-income black and white adults from 2002 to 2009. Participants for this analysis were 11,948 women who were postmenopausal at enrollment, had no history of HF, and were on Medicaid or Medicare. HF events were ascertained using ICD-9 codes 428.x via linkage of the cohort with CMS Research Identifiable Files through December 31, 2010. Early menopause was defined as self-reported age at menopause less than 45 years. Hazard ratios (HRs) and 95% confidence intervals (CIs) were computed from multivariable Cox regression models, overall and by race, adjusting for demographic, lifestyle, and reproductive factors, including reason for menopause. RESULTS/ANTICIPATED RESULTS: At baseline, mean age was 58±9 years, and 65% of participants were black. Among women with early menopause, 76% (n = 4,836) had menopause due to hysterectomy or oophorectomy. In women with later menopause, 74% (n = 4,102) reported natural menopause. During a median follow-up of 5.0 years (range 3.1-6.7), 2,157 incident HF events occurred. Compared with women with later onset of menopause, those with early menopause had increased HF risk (HR: 1.27, 95% CI: 1.10–1.47). Risk of HF associated with early menopause was similar in white and black women (p-value for interaction: 0.13). DISCUSSION/SIGNIFICANCE OF IMPACT: In this largely low-income population, early menopause was associated with an increased risk of developing HF. Women with early menopause represent a potential target population for future interventions to decrease risk of HF and cardiovascular risk factors.

